# Childhood cancer incidence and survival trends in Estonia (1970–2016): a nationwide population-based study

**DOI:** 10.1186/s12885-019-6510-7

**Published:** 2020-01-10

**Authors:** Keiu Paapsi, Aleksei Baburin, Sirje Mikkel, Margit Mägi, Kadri Saks, Kaire Innos

**Affiliations:** 1 Department of Epidemiology and Biostatistics, National Institute for Health Development, Hiiu 42, 11619 Tallinn, Estonia; 2 Clinic of Haematology and Oncology, Tartu University Hospital, Tartu, Estonia; 3 Estonian Cancer Registry, National Institute for Health Development, Tallin, Estonia; 4 Department of Oncology and Haematology, Clinic of Paediatrics, Tallinn Children’s Hospital, Tallinn, Estonia

**Keywords:** Childhood cancer, Incidence, Survival, Population-based, Cancer registry, ICCC-3

## Abstract

**Background:**

Childhood cancers represent a small proportion of all cancers but are still a major public health problem. The study analysed long-term trends in childhood cancer incidence and survival in Estonia in relation to societal and health care transition.

**Methods:**

Data on all malignant tumours, diagnosed in children aged 0–14 during 1970–2016, were derived from the Estonian Cancer Registry. Age-standardised (World standard) incidence rates were calculated by ICCC-3 site groups and joinpoint regression was used to estimate annual percentage change (APC) for incidence trends. Cohort and period approach were used to estimate 5-year survival. Internal age standardisation was applied.

**Results:**

A total of 1628 incident cancer cases were diagnosed during the study period and overall incidence increased significantly at a rate of 0.5% per year. Significant increases were seen for neuroblastoma and germ cell tumours, for lymphoid leukemias and some CNS sub-sites. At the same time, decline in incidence was seen in almost all subgroups of unspecified neoplasms. The overall 5-year survival improved from 24% in 1970–1979 to 73% in 2010–2016, with the largest changes occurring in the 1990s and 2000s. For many sites, survival increase thereafter has been marginal.

**Conclusion:**

In this first comprehensive population-based study of childhood cancer incidence and survival in Estonia, long-term trends are shown in the context of societal and health care changes. Even though the increasing incidence of some sites may, at least partially, be explained by improved diagnostics reflected in the decreased incidence of unspecified neoplasms, the overall cancer incidence in children seems to be rising. Rapid progress in diagnosis and care have improved childhood cancer survival immensely, but deficit in Estonia persists compared to other European countries. Results of the study accentuate the need for a more in-depth analysis of clinical data, but also for the prioritization of childhood cancer in Estonia, to ensure access to standard care and innovative treatments.

## Background

In Estonia, childhood cancer is the third leading cause and the main disease-related cause of death in children under the age of 15. Estonia has witnessed societal and political transition during the past decades and has moved from the Soviet health care system into a more modern and centralised system that has brought along tremendous changes in childhood cancer management. No studies, to this date, have been conducted in Estonia to analyse long-term time-trends of childhood cancer incidence and survival nor the impact of the above-mentioned changes. But the increasing number of patients [1] and better outcomes seen in clinical practice, and inferior survival compared to other countries seen in international studies [2, 3], clearly indicated the need for it. Furthermore, as more and more children survive and remain at risk of developing secondary malignancies, chronic diseases and many other treatment emergent late effects, new challenges are imposed on health care. Therefore, monitoring incidence and survival trends is crucial to inform public health policy and health services.

The aim of the study was a) to analyse long-term childhood cancer incidence and survival trends in Estonia by sex, age and site group, b) to examine these trends in the context of the advances in cancer treatment through changes in the political order and in the health care system.

## Methods

The population-based, nation-wide Estonian Cancer Registry (ECR) holds data on all incident cancer cases diagnosed in Estonia since 1968 (population 1.3 million according to the 2011 census). All malignant, in situ*,* benign tumours and tumours of uncertain or unknown behaviour of brain and central nervous system as well as of the endocrine organs, located in the area of the brain and other tumours of lymphoid, haematopoietic and related tissues, are registered. ICD-O-3 coding is used for all cases registered in the ECR.

Data on all primary malignant tumours diagnosed between 1970 and 2016 in children aged 0–14 years were derived from the ECR. As non-malignant CNS tumours are registered only starting from 1998, these cases were not included in this study. Age at diagnosis was grouped into three categories: 0–4, 5–9, 10–14 years. Cancer sites were grouped as defined by the third edition of the International Classification of Childhood Cancer (ICCC-3) on the basis of the ICD-O-3 topography and morphology [4]. Follow-up for vital status from the date of diagnosis until date of death, emigration or December 31, 2016 was performed by the ECR using unique personal identification numbers or name and date of birth during earlier periods. Cases are first linked with the Estonian Causes of Death Registry to identify deaths, followed by linkage of alive cases to the Estonian Population Registry to identify emigration. Percentage of microscopically verified cases, percentage of death certificate only cases (%DCO) and percentage of cases discovered at autopsy were used as data quality indicators.

Incidence rates were calculated as the number of new cases per million persons per calendar year. Population data were derived from Statistics Estonia. In order to have sufficient number of cases, the study period was divided into two intervals – 1970 to 1994 and 1995 to 2016. Age-standardised rates were calculated using the weights of the World standard population [5]. Incidence trends were calculated by sex, age group (0–4, 5–9, 10–14) and cancer site, using Joinpoint Regression Program 4.6.0.0 (April 2018; Statistical Methodology and Applications Branch, Surveillance Research Program, National Cancer Institute) and presented as annual percentage change (APC) and corresponding 95% confidence intervals (CI). APC over the whole study period by ICCC-3 site groups is presented in the main table. Joinpoints for subsites or age groups are shown in the text. Additional sensitivity analysis was carried out for leukemias starting from 1985 onwards (to investigate incidence trends after it became possible to distinguish between ALL and AML).

For survival analysis, the study period was divided into five periods: 1970–1979, 1980–1989, 1990–1999, 2000–2009, 2010–2016. 5-year observed survival with 95% CI were calculated by ICCC-3 site groups and time periods using the Kaplan-Meier method. We used cohort approach for the first four periods and period approach for the latest, as this allows the prediction of survival for children with yet incomplete follow-up [6]. Internal age standardisation (to the latest period) was applied. DCO and autopsy cases were excluded from the survival analysis.

Statistical analysis was performed with STATA 14.2 (StataCorp, College Station, Texas, USA). The study protocol was approved by the Tallinn Medical Research Ethics Committee.

## Results

In total, 1628 incident cases of childhood cancer were diagnosed in Estonia during 1970–2016 (Table 1). Boys were diagnosed more frequently, giving a male to female ratio of 1.2 and nearly half of the cases were diagnosed in the youngest age group. Data quality in the ECR improved significantly over the study period. The proportion of cases verified microscopically increased and only 0.4% of the cases were reported via death certificate only. Since 2007, all cases have been diagnosed in life.

**Table 1:** Malignant childhood cancer cases diagnosed in Estonia, 1970–2016

	Cases	(%)	MV (%)	DCO (%)	Autopsy (%)
Total	1628	100	91.8	0.4	4.4
Sex					
Boys	891	54.7	91.6	0.5	4.5
Girls	737	45.3	92.1	0.3	4.2
Age (years)					
0–4	714	46.3	92.0	0.3	6.1
5–9	415	25.5	91.8	0.7	3.4
10–14	460	28.3	91.5	0.2	2.4
Period of diagnosis					
1970–1974	184	11.3	76.6	0.5	12.0
1975–1979	162	10.0	87.0	0.6	8.0
1980–1984	209	12.8	87.1	0.0	8.6
1985–1989	199	12.2	91.0	0.0	5.5
1990–1994	212	13.0	95.8	1.4	1.9
1995–1999	180	11.1	96.7	0.0	1.1
2000–2004	152	9.3	98.0	0.7	0.0
2005–2009	124	7.6	98.4	0.0	0.8
2010–2016	206	12.7	98.1	0.0	0.0

Abbreviations: *MV* microscopically verified, *DCO* death certificate only

### Incidence

The number of cases, incidence rates, trends described by APC are presented in Table 2 by ICCC-3 site groups. No joinpoints were found for the main ICCC-3 site groups, therefore APC over the whole study period is presented in the table. The age-standardised incidence of all sites for 1970–2016 was 122.8 per million and the rate increased significantly over the study period (APC 0.5%). Leukemias were diagnosed most frequently, followed by CNS tumours and lymphomas (ASIR 40.6, 24.0, 16.9 per million, respectively). Incidence rate was the lowest for other and unspecified malignant neoplasms (1.7 per million).

**Table 2:** Age-standardised incidence rates of childhood cancer by ICCC3 site-groups (malignant only), Estonia 1970–2016

	Total cases	%	Average annual cases	ASIR/million 1970–1994	ASIR/million 1995–2016	APC (%) (1970–2016)	95% CI
All sites	1628	100	35	122.2	138.1	**0.5**	**0.1–0.9**
I Leukemias, myeloproliferative diseases, and myelodysplastic diseases	506	31.1	11	39.5	42.1	0.2	−0.7–1.0
a. Lymphoid leukemias	302	18.6	6	20.0	31.4	**2.1**	**0.1**–**4.0**
b. Acute myeloid leukemias	72	4.4	2	4.3	7.4	1.8	−1.2–5.0
c. Chronic myeloproliferative diseases	6	0.4	0	0.3	0.9	NC	NC
d. Myelodysplastic syndrome and other myeloproliferative diseases	7	0.4	0	0	1.6	NC	NC
e. Unspecified and other specified leukemias	119	7.3	3	15.0	0.7	**−11.5**	**−13.4;-9.6**
II. Lymphomas and reticuloendothelial neoplasms	232	14.3	5	16.8	17.2	−0.1	−1.2–1.0
a. Hodgkin lymphomas	95	5.8	2	7.7	5.1	−1.5	−3.6–0.7
b. Non-Hodgkin lymphomas (except Burkitt lymphoma)	93	5.7	2	7.4	6.0	−1.0	−2.8–0.9
c. Burkitt lymphoma	17	1.0	0	0	3.3	**8.8**	**7.4–10.2**
d. Miscellaneous lymphoreticular neoplasms	15	0.9	0	0.5	2.4	NC	NC
e. Unspecified lymphomas	12	0.7	0	1.2	0.4	−2.0	−5.4–1.5
III. CNS and miscellaneous intracranial and intraspinal neoplasms (malignant only)	312	19.2	7	22.0	27.5	0.9	−0.5–2.3
a. Ependymomas and choroid plexus tumors	43	2.6	1	1.9	6.2	**3.3**	**1.5–5.1**
b. Astrocytomas	110	6.8	2	7.7	9.0	1.1	−2.7–5.0
c. Intracranial and intraspinal embryonal tumors	78	4.8	2	4.9	8.1	2.4	−0.2–5.1
d. Other gliomas	16	1.0	0	0.7	2.0	**3.5**	**1.2–5.8**
f. Unspecified intracranial and intraspinal neoplasms	64	3.9	1	6.7	1.9	**−5.1**	**−8.4;-1.7**
IV. Neuroblastoma and other peripheral nervous cell tumors	91	5.6	2	6.3	10.4	**2.3**	**0.0–4.7**
a. Neuroblastoma and ganglioneuroblastoma	89	5.5	2	6.1	10.4	**2.5**	**0.3–4.7**
V. Retinoblastoma	41	2.5	1	3.4	4.0	−0.2	−3.4–3.2
VI. Renal tumors	132	8.1	3	12.6	9.1	−0.8	− 3.0–1.4
a. Nephroblastoma and other nonepithelial renal tumors	127	7.8	3	11.9	9.1	−0.6	−2.7–1.5
VII. Hepatic tumors	26	1.6	1	1.9	2.9	1.1	−1.7–4.1
a. Hepatoblastoma	18	1.1	0	1.0	2.7	NC	NC
VIII. Malignant bone tumors	71	4.4	2	4.6	5.5	0.3	−2.6–3.2
a. Osteosarcomas	27	1.7	1	1.7	2.2	−0.6	−3.6–2.5
c. Ewing tumor and related sarcomas of bone	25	1.5	1	1.1	2.6	3.2	−0.6–7.1
e. Unspecified malignant bone tumors	12	0.7	0	1.1	0.3	**−3.3**	**−4.8;-1.7**
IX. Soft tissue and other extraosseous sarcomas	81	5.0	2	5.8	7.2	1.3	−0.0–2.6
a. Rhabdomyosarcomas	32	2.0	1	1.8	3.9	3.0	−0.5–6.7
b. Fibrosarcomas. peripheral nerve sheath tumors. and other fibrous neoplasms	12	0.7	0	1.1	0.6	0.4	−2.1–2.9
d. Other specified soft tissue sarcomas	26	1.6	1	1.6	2.5	1.5	−1.4–4.4
e. Unspecified soft tissue sarcomas	10	0.6	0	1.2	0.3	−2.0	−4.9–1.0
X. Germ cell tumors, trophoblastic tumors, and neoplasms of gonads	75	4.6	2	4.7	8.3	**2.5**	**0.2–4.9**
a. Intracranial and intraspinal germ cell tumors	8	0.5	0	0.1	1.3	**5.1**	**2.1–8.1**
b. Malignant extracranial and extragonadal germ cell tumors	34	2.1	1	2.0	4.6	**3.4**	**1.1–5.7**
c. Malignant gonadal germ cell tumors	26	1.6	1	1.9	2.2	0.7	−2.2–3.7
XI. Other malignant epithelial neoplasms and malignant melanomas	41	2.5	1	2.3	3.5	1.4	−0.8–3.7
b. Thyroid carcinomas	10	0.6	0	0.2	1.3	3.7	−0.9–8.7
d. Malignant melanomas	12	0.7	0	0.8	0.8	−0.2	−2.8–2.5
f. Other and unspecified carcinomas	12	0.7	0	0.9	0.8	0.3	−2.2–2.8
XII. Other and unspecified malignant neoplasms	20	1.2	0	2.3	0.6	**−2.4**	**−3.9;-0.9**
b. Other unspecified malignant tumors	17	1.0	0	2.0	0.4	**−3.4**	**−4.5;-2.3**

Abbreviations: *ICCC-3* international classification of childhood cancer, third edition; *ASIR* age-standardised incidence rate, *APC* annual percentage change, *CI* confidence intervals, *NC* not calculated

Table shows all diagnostic groups with > 5 patients diagnosed over the study period. Values that are statistically significant are marked in bold

Leukemias showed a stable trend overall (APC 0.2%), with a significant increase for lymphoid leukemias (APC 2.1%) and significant decrease for unspecified leukemias (APC − 11.5%). By age groups, a significant increase was seen in lymphoid leukemias in age group 5–9. The increase was steeper in 1970–1985 (APC 7.9, 95% CI 3.2–12.8), followed by a steadier pace (APC 1.8, 95% CI 1.2–2.5). Leukemias increased overall for girls, but not for boys. Girls aged 10–14 have experienced a fluctuating trend – a decrease in 1970–1985 and 2000–2010 (APC -6.0, 95% CI − 7.4 to − 4.5; and − 3.3, 95% CI − 5.9 to − 0.6) and an increase of 10.0% (95% CI 7.1–13.1) in 1985–2000.

The incidence of lymphomas remained unchanged (APC − 0.1%), but there was a significant increase in the incidence of Burkitt lymphoma, based on a very small number of cases. Overall, there was no increase in the incidence of CNS tumours, but a significant increase was seen for boys (APC 1.5, 95% CI 0.2–2.8). The significant trend seen for ependymomas and choroid plexus tumors was due to a significant rise by 7.2% (95% CI 4.7–9.7) in the youngest age group. At the same time, the incidence of unspecified intracranial and intraspinal neoplasms decreased significantly. Neuroblastoma and other peripheral nervous cell tumors (group IV) incidence increased significantly by 2.3%. The latter increase can be attributed to age group 0–4 (APC 3.1%, Additional file 1: Table S1). Within this age group, incidence increased for boys (APC 2.9, 95% CI 0.6–5.2), and for subsite neuroblastomas and ganglioneuroblastomas (APC 3.3, 95% CI 1.4–5.1). The increase was more pronounced in age group < 1 year compared to age group 1–4 (APC 4.0, 95% CI 1.4–6.6 vs 2.9, 95% CI − 0.3–6.1, respectively). Incidence of retinoblastomas, which were mainly diagnosed in the youngest age group of 0–4, remained stable over the study period (APC − 0.2%, Table 2), but an upward trend was seen for boys aged 0–4 (APC 2.7, 95% CI 0.1–5.5). For renal tumors, the overall steady incidence (APC − 0.8%) has shown a decrease in boys since 1990 (APC 2.9, 95% CI − 5.3 to − 0.3). The incidence of hepatoblastoma, the main malignancy among hepatic tumors increased rapidly from 1985 to 2000 (APC 14.1, 95% CI 2.3–27.3). Among malignant bone tumours, a significant decrease was seen for unspecified tumours. Incidence of soft tissue and other extraosseous sarcomas increased in boys (APC 2.4, 95% CI 1.1–3.8). Significant increase in germ cell and gonadal tumors by 2.5% was caused by a rise in 0–4-year olds (APC 2.7%, Additional file 1: Table S1). The number of unspecified neoplasms has decreased significantly.

**Fig. 1:**
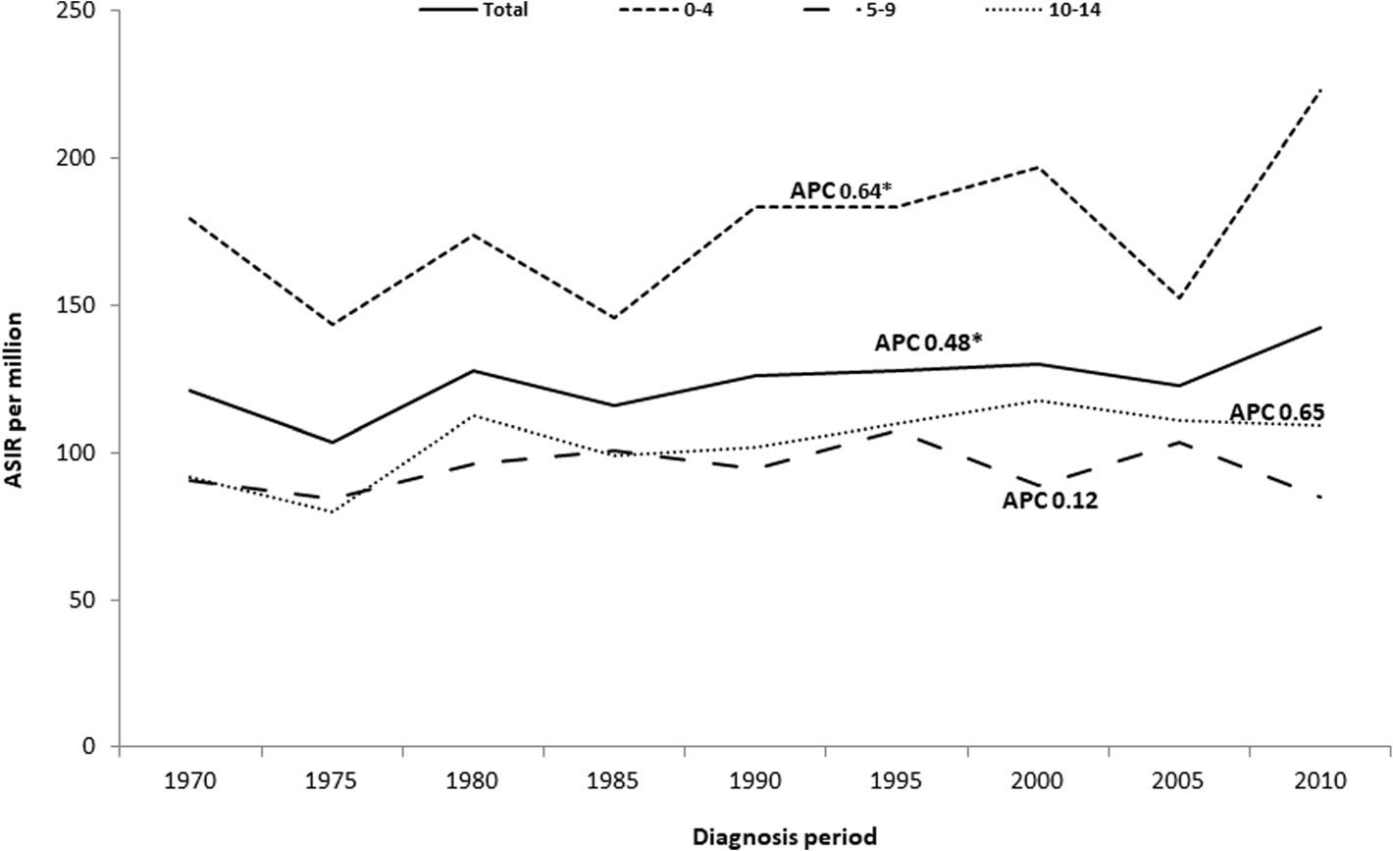
Age-specific childhood cancer incidence rates (all sites combined, per million) in Estonia by period of diagnosis. (*The APC is significantly different from zero at alpha = 0.05)

For both sexes all sites combined, age-specific incidence increased in all age groups, but significant rise was seen only for the age group 10–14 (APC 0.6, 95% CI 0.1–1.2) (Fig. 1).

### Survival

Table 3 shows the 5-year survival estimates by ICCC-3 site groups and time periods. Survival increased most markedly from 1980 to 1989 to 1990–1999 and continued to rise through the following decades at a slower pace. For all malignant neoplasms combined, survival rates increased from 23.8% in 1970–1979 to 73.0% by 2010–2016. Increase has been most substantial for leukemias and renal tumors (from below 10% to over 80%). Acute lymphoid leukemia (ALL) survival increased the most from 1985 to 1989 (32.9, 95% CI 18.0–48.7) to 1990–1994 (53.6, 95% CI 39.2–66.1) and from 2000 to 2004 (67.0, 95% CI 49.0–79.8) to 2005–2009 (86.0, 95% CI 70.2–93.7). Acute myeloid leukemia (AML) survival increased the most from 1985 to 1989 (0%) to 1990–1994 (40, 95% CI 5.0–75.3), 1995–1999 (42.9, 95% CI (9.8–73.4) to 2000–2004 (55.4, 95% CI 14.9–83.3) and from 2000 to 2004 to 2005–2009 (73.1, 95% CI 39.5–90.0).

**Table 3:** Childhood cancer survival by ICCC3 site groups and time periods, Estonia 1970–2016

ICCC3	1970–1979			1980–1989			1990–1999			2000–2009			2010–2016		
	5-year survival	95% CI		5-year survival	95% CI		5-year survival	95% CI		5-year survival	95% CI		5-year survival	95% CI	
		Low	High		Low	High		Low	High		Low	High		Low	High
All sites (malignant)	23.8	19.2	28.6	30.0	25.4	34.8	52.5	47.2	57.5	69.4	63.3	74.7	73.0	66.2	78.7
I. Leukemias, myeloproliferative diseases, and myelodysplastic diseases	7.5	3.5	13.4	19.6	12.6	27.6	51.9	42.0	61.0	71.6	61.4	79.5	83.8	72.0	90.9
Ia. Lymphoid leukemias	7.4^a^	1.3	21.0	21.8	13.0	32.1	56.4	45.3	66.1	75.0	62.9	83.6	83.3	69.4	91.3
Ib. Acute myeloid leukemias	0^a^	–	–	5.8^a^	0.4	23.2	41.7^a^	15.3	66.5	60.3	34.7	78.5	81.0	43.0	94.9
II. Lymphomas and reticuloendothelial neoplasms	44.8	27.5	60.6	44.4	25.0	62.1	60.0	40.4	74.9	83.9	51.0	95.5	90.2	72.6	96.7
IIa. Hodgkin lymphomas	82.7	69.8	90.4	57.8	15.8	85.0	92.6	83.5	96.8	100	–	–	100	–	–
IIb. Non-Hodgkin lymphomas (except Burkitt lymphoma)	28.7	11.8	48.3	38.2	14.4	62.0	40.5	16.5	63.5	73.0	20.1	94.0	100	–	–
III. CNS and miscellaneous intracranial and intraspinal neoplasms (malignant only)	25.4	12.7	40.2	22.4	12.9	33.6	49.6	38.4	59.8	59.8	44.6	72.0	49.5	32.4	64.5
IV. Neuroblastoma and other peripheral nervous cell tumors	12.5^a^	0.7	42.3	47.4^a^	24.4	67.3	32.3^a^	16.9	48.6	40.0^a^	16.5	62.8	54.4	24.4	76.9
0–4 years^b^	16.7	2.4	42.3	42.9	23.9	60.6	42.9	27.3	57.6	46.2	25.9	64.2	54.2	24.3	76.7
V. Retinoblastoma	74.1^a^	39.1	90.9	70.0^a^	32.9	89.2	100^a^	–	–	66.7^a^	5.4	94.5	100	–	–
VI. Renal tumors	9.2^a^	1.7	25.0	27.3^a^	14.3	42.1	55.6^a^	38.1	69.9	80.0^a^	50.0	93.1	90.0	47.3	98.5
VII. Hepatic tumors	0^a^	–	–	16.7^a^	0.8	51.7	40.0^a^	5.2	75.3	0^a^	–	–	100^a^	–	–
VIII. Malignant bone tumors	15.4^a^	2.5	38.8	18.2^a^	5.7	36.3	50.0^a^	25.9	70.1	80.0^a^	40.9	94.6	64.3	25.3	86.8
IX. Soft tissue and other extraosseous sarcomas	50.0^a^	18.4	75.3	19.6^a^	6.0	38.9	35.3^a^	14.5	57.0	73.3^a^	43.6	89.1	58.9	27.7	80.4
X. Germ cell tumors, trophoblastic tumors, and neoplasms of gonads	25.0^a^	6.0	50.5	37.5^a^	15.4	59.8	45.5^a^	16.7	70.7	76.2^a^	51.9	89.3	37.0	11.3	63.6
XI. Other malignant epithelial neoplasms and malignant melanomas	75.0^a^	31.5	93.1	55.6^a^	20.4	80.5	87.5^a^	38.7	98.1	100^a^	–	–	100	–	–
XII. Other and unspecified malignant neoplasms	14.3^a^	0.7	46.5	0^a^	–	–	0^a^	–	–	0^a^	–	–	100	–	–

Abbreviations: *ICCC-3* international classification of childhood cancer, third edition, *CI* confidence intervals. ^a^ Not age standardised, due to small number of cases. ^b^ as neuroblastomas are mainly diagnosed in age group 0–4, survival for this age group is presented in table for international comparisons

**Fig. 2:**
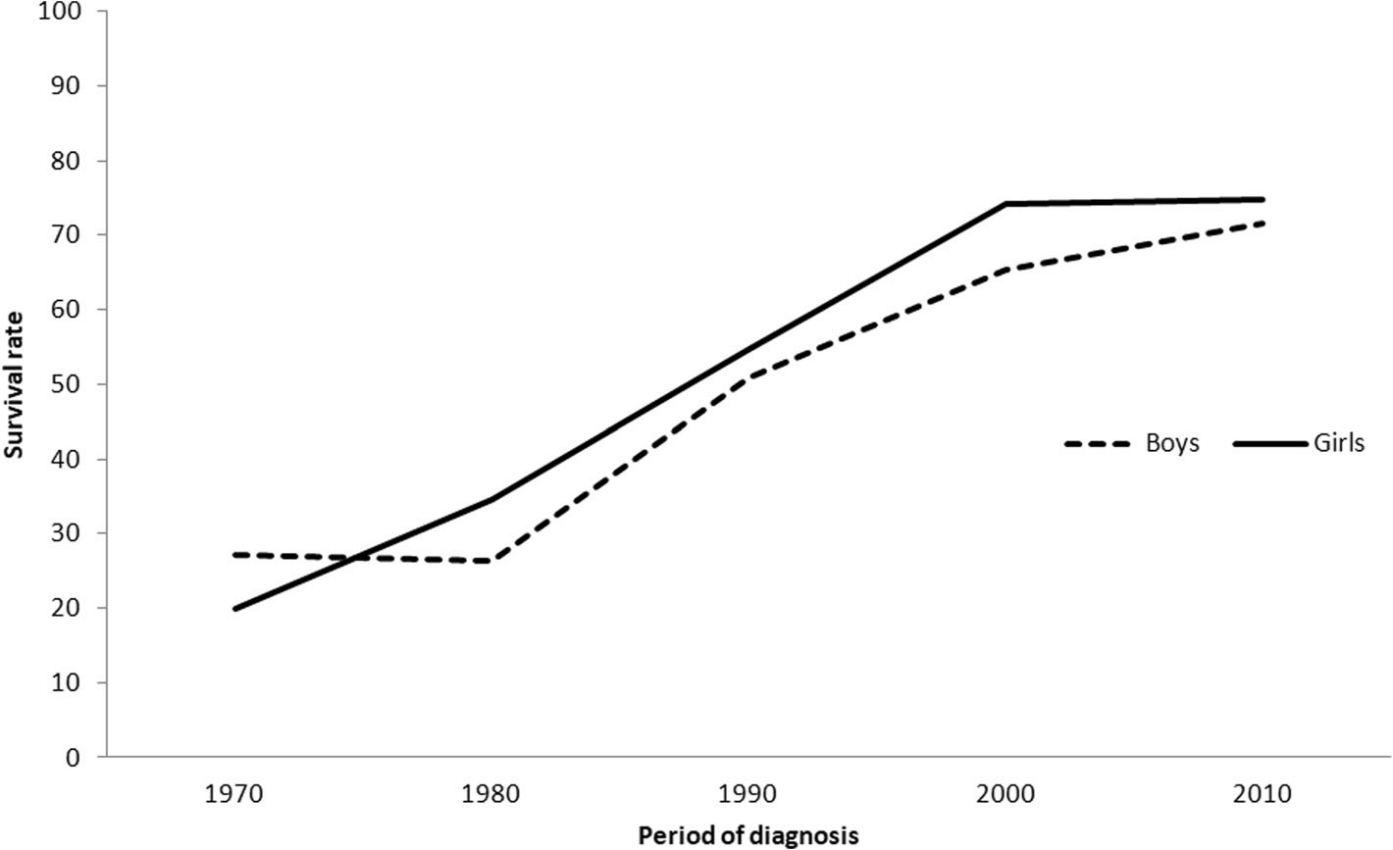
5-year childhood cancer survival in Estonia by sex and time period

Survival has increased for both sexes (Fig. 2), being initially higher for boys, but increasing more rapidly for girls since 1980. First two decades did not see any changes in the 5-year survival in boys, whereas the 5-year survival in girls already improved from 19.8% (95% CI 13.7–26.7) to 34.6% (95% CI 27.3–42.0). Increase was most substantial in boys from 26.4% in 1980–1989 (95% CI 20.6–32.6) to 50.8% in 1990–1999 (95% CI 43.3–57.8). Improvements in 5-year survival in girls occurred through the first three decades, but after a considerable increase, has now been stable for the last two decades (74.1 and 74.8%, accordingly). Survival in boys has continued to increase, reaching 71.6% in the latest period (95% CI 62.3–79.0). The site distribution differed significantly between boys and girls (*p* = 0.001).

## Discussion

This population-based study spanning nearly 50 years showed increasing incidence and survival of childhood cancer in Estonia. Survival improvements varied by cancer site and were most substantial before the 2000s.

Cancer registration began in the Soviet Union in 1953. At the very early stages of cancer registration no standard disease classification nor age groups were used, data was published annually, but never corrected. The ECR dates back to 1968. This nation-wide population-based registry holds data for adult and childhood cancer cases, including benign CNS tumours since 1998. Reporting to the registry is mandatory for all physicians who diagnose or treat reportable tumours. Case registration according to international rules was implemented step-by-step starting with the introduction of ICD-O morphology section in 1978 and the topography section in 1983. The 3rd edition of TNM classification of Malignant Tumors was translated into Estonian in 1980. ECR data was first included in the Cancer Incidence in Five Continents volume 6, published in 1992. All cases have been retroactively coded to ICD-O-3. Currently, the ECR follows international definitions and rules, including those for multiple primaries, issued by the European Network of Cancer Registries and the International Association of Cancer Registries [7], for reporting incidence and survival.

Good data quality has been shown for adult cancers [8]. According to a previous study [9], the estimated completeness of reporting of childhood cancer cases was 89.5% (81.2% for hematologic malignancies and 95.3% for solid tumors). As a result of the data quality study, all missing cases starting from 2000 were added to the cancer registry and regular linkage with all treating hospitals was established. Thus, we believe that the disruption of registry practices in the 2000s [10] has no further impact on case registration and the estimated completeness of childhood cancer is now close to 100%. The validity of childhood cancer data has improved markedly over time – there were only two autopsy cases and no DCO cases since 2003. The proportion of microscopically verified cases has increased throughout the study period, now reaching 98%. ECR data has been included in major childhood cancer studies in Europe [2, 11], being a good indication of the data quality.

Methods for diagnosing improved during the study period. Ultrasound became available for prenatal screening since the early 1990s (first ever used in 1984), MRI in the 2000s. Most recent changes in histology were introduced in 2010 (molecular pathology).

The main strengths of the study were nation-wide population-based cancer registry coverage with complete follow-up and high data quality, enabling the evaluation of incidence and survival trends over a 47-year period, with the most recent data for patients diagnosed in 2016. The main limitations of this study were the scarcity of cases (causing a fluctuation in trends and instability of survival estimates) and the quality of morphological verification in earlier periods that might have caused some misclassification of cases.

### Incidence

The aetiology of childhood cancers is still in large part unknown. Many hypotheses have been made about different environmental, parental and gestational factors and some genetic causes have been established, but the scarcity of data and studies make it difficult to draw definite conclusions [12]. Childhood cancer incidence in Estonia increased during 1970–2016 at a rate of 0.48% per year, comparable to rates in Europe and Eastern-Europe in 1991–2010 (0.54 and 0.50% respectively). Even though hypothesised that studies, where data was analysed starting from the 1970s, may see increase in rates in large part due to the rise in the earlier decades [13] (caused by improved diagnostics, decreased overall child mortality), we saw the opposite. The overall age-standardised incidence of childhood cancers in Estonia for the first half of the study period was notably lower than the European average (122.2 vs 140.0 per million) but reached the European rate for 1991–2010 in the second half (138.1 vs 137.5 per million). Regarding the fact that our study dates back to 1970, we can assume that in some part the increase in incidence can be explained by improvements in diagnosing and registration. Diagnostic drift must also be taken into consideration, especially for cancer sites where overall incidence for the site group remains stable through time but changes occur among subsites. Other possible explanation for incidence increase, especially in an atypical age group may be a delay in diagnosis, highly probable for that time period. On the other hand, not all can be attributed to improved diagnostics. Rise in incidence for some sites (e.g neuroblastomas, soft tissue sarcomas etc) and all sites combined, indicates an actual rise probably due to the risk factors mentioned above. The same applies for competing risk theory, as infant and child mortality have decreased since the 1970s.

Leukemias, the most commonly diagnosed malignancies, showed a slight increase in incidence. ASIR in Estonia was lower in both periods than shown for Europe in ACCIS studies, for the periods 1970–1999 and 1991–2010 (39.5 vs 44.8 and 42.1 vs 46.9 per million). Rate for Europe has increased steadily by 0.6% per year in both periods [14, 15], whereas Estonian average increase of 0.2% per year for the whole period is more characteristic to Eastern-European trend (increase of 0.3% per year for 1978–1997 [16] and 1% in 1991–2010) [14]. Since there was a substantial decrease in unspecified leukemias a restricted analysis was done starting from 1985 onwards when the proportion of unspecified cases became negligible to control whether the increase was solely due to improved diagnostics. Even though non significant, incidence increased from 1985 onwards for ALL (APC 1.4, 95% CI -0.6–3.5), AML (APC 2.4, 95% CI − 3.4–8.5) and leukemias combined (APC 1.4, 95% CI -0.2–3.0), referring to other underlying causes (data not shown). Several environmental and parental lifestyle, residential and occupational exposures have been associated with pediatric leukemias [16] but among known risk factors, high birth weight and increased maternal age [17] could be possible reasons here [18]. Some studies show that early day-care attendance is protective against leukemia [19, 20] due to exposure to infections. Starting from early 2000s, children in Estonia attend day-care less and start at a later age, as mothers now have the option for prolonged and paid leave.

Incidence of lymphomas is high in Estonia (16.9 per million for 1970–2016), as has been described also for other Eastern-European countries [14]. Rate is increasing in Europe by 0.26% per year, but stable in Estonia and declining in Eastern-Europe (APC − 0.1% and − 1.32%, respectively) [14]. Even though the overall rate is relatively stable, an immense increase by 8.8% was seen for Burkitt lymphomas, which generally contribute proportionally lower to lymphoid malignancies [16]. Similar trend, but at a much slower rate of 1.4% per year, was seen for Spain in 1983–2007 [21], while a decreasing trend was shown in Canada (APC − 2.54% for 1992–2010) [22]. As lymphomas tend to be more frequent in less developed countries and of infectious origin, our finding is difficult to reason. Since the rise has been more recent, it could partly be explained by improved diagnostics and misclassification, as the incidence of all other subgroups have declined. Classification has changed for miscellaneous lymphoreticular neoplasms (IId). In previous ICD-O classifications Langerhans cell histiocytosis was not a reportable disease. ICD-O-3 includes codes for both malignant and uncertain disease. Thus, increase in incidence seen in our data could be caused by the changes in classification.

Likewise, the incidence of unspecified neoplasms of CNS decreased significantly, while the overall trend remained relatively stable. Therefore, the observed increase in the incidence of ependymomas (IIIa) and other gliomas (IIId) needs to be interpreted with caution. In the earlier periods, the incidence rates for ependymomas and gliomas were lower in Estonia compared to Eastern-Europe (1.9 per million in Estonia compared to 3.3 per million in Eastern-Europe for ependymomas and 0.7 vs 2.2 per million for gliomas, respectively) [23]. More recent incidence rates for ependymomas are now higher in Estonia than in the Nordic countries and France (6.2 vs 4.2 vs 3.8, per million, respectively), whereas incidence rate for other gliomas has remained lower – 2.0, 3.5 and 4.9 per million, respectively [24, 25]. The absence of overall trend in incidence is most probably due to the changes in tumor classification and the tendency in earlier years to record benign cases as malignant.

Neuroblastoma incidence in Estonia increased significantly over the study period at a rate of 2.3% per year due to a significant increase in the 0–4 age group (APC 3.1%) and subsite IVa (AAPC 2.5%). Incidence of subsite IVa increased more for age group < 1 (APC 4.0, 95% CI 1.4–6.6) compared to ages 1–4 (APC 2.7, 95% CI -0.3–6.1) (data not shown). A similar overall trend was seen for Eastern-Europe in 1978–1997 (AAPC 2.7%) [26]. For other countries, the incidence seems to increase at a slower pace, for example in Canada (APC 0.74% in 1992–2010) [22]. Since we do not see a distinct leap in the incidence in any of the time periods, we can not conclude that the increase is solely attributable to improvements in diagnostics, e.g. wider use of ultrasound (1990s), MRI (2000s) or molecular pathology (2010), which has been shown to improve the differentiation of neuroblastomas from other small round cell tumors like rhabdomyosarcoma and Ewing’s sarcoma [27], but the latter two also show an increasing trend in Estonia. Several risk factors, such as maternal oral contraceptives or sex hormones use during pregnancy, a shorter gestational duration, and maternal alcohol consumption during pregnancy [28], have been debated as possible causes, but need to be analysed further.

Hepatic tumors show an increasing trend in Estonia and elsewhere [22, 26]. Even though non-significant, the rise was noticeable in the youngest age group, with almost half of the cases diagnosed in infants less than 1 year of age (data not shown). In these cases, perinatal exposures and decreased premature infant mortality could be postulated causes [29]. Almost 90% of the cases diagnosed in age group 0–4 were hepatoblastomas, which is associated with low birth weight [30]. The proportion of low birth weight babies has remained the same in Estonia, but their survival has increased over time [18].

The incidence of both intracranial and extracranial germ cell tumors increased significantly. The incidence of intracranial germ cell tumors is in accordance with the rate in Europe (about 1.0 per million) [31] and in Germany (1.2 per million, and also increasing), but the incidence of extracranial germ cell tumors is higher in Estonia and showing a reversed trend compared to Germany (decreasing at 0.1%) [32]. We see two peaks in incidence, first one falls to the mid 1980s, when certain biochemical methods and computer tomography were introduced [33], and another spike more recently, which could refer to changes in clinical and treatment practices for childhood ovarian tumors [32]. A more detailed analysis of clinical data by subsite is warranted.

### Survival

Estonia regained its independence in 1991, at midpoint of the study period. The latter resulted in major reforms, where solidarity-based health insurance was established and the health care was changed from the state-run Semashko [34] system to a more Western one. Integrated and hierarchically organised system, which was communicable diseases and primary care oriented, was replaced by decentralized model funded through social insurance.

**Fig. 3:**
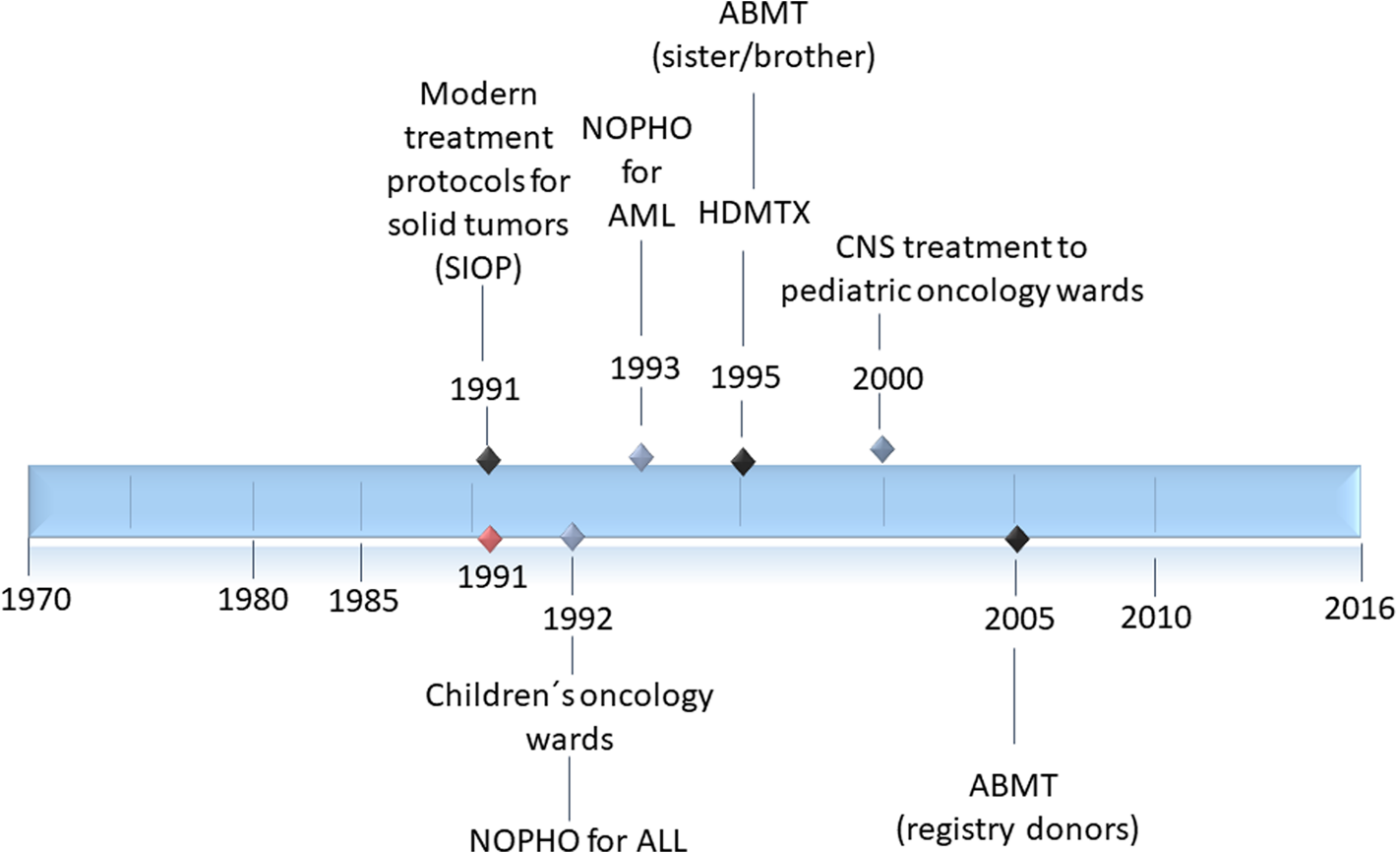
Time-line of treatment and health care organisation in Estonia from 1970 to 2016. Year 1991 marks the regaining of independence

Childhood cancer treatment in Estonia in 1970s to 1990s was scattered between departments and institutions. At that time, hematologic malignancies were treated in the general pediatric departments of two major hospitals in Tallinn and Tartu. Solid tumors were operated in the surgical departments together with adults until 1979, when it was partly moved to the surgical department of Tallinn Children’s Hospital. Radiation was the main treatment and chemotherapy was used scarcely at that time. Even though the centralisation of childhood cancer care began in 1992 when the first pediatric oncology wards were opened, children were still treated together with adults for a long time. CNS treatment was gradually brought to pediatric oncology departments in 1995–2000, whereas low grade tumors are still partly managed at adult centres. The arrival of first European treatment protocols in 1991 for solid tumors (SIOP, Société internationale d’oncologie pédiatrique) and 1992 for hematologic malignancies (NOPHO, Nordic Society for Pediatric Hematology and Oncology) set the base for twinning with European oncology organisations, enabling Estonia to reach new information and treatment protocols faster (Fig. 3). NOPHO protocols for ALL and AML were at first hand used partially in 1992–2005. More advances followed with the introduction of high dose methodrexate treatment (HDMTX) and bone marrow transplant from sibling donors in 1995. In 2000, when CNS treatment was transferred to children’s oncology department, modern protocols and chemotherapy became available. Starting from 2008 for ALL and 2012 for AML Estonian patients are included in NOPHO clinical trials. Even though Estonia has never participated in clinical trials for solid tumors, standard treatment protocols are used according to guidelines. Over time, the main issues of managing childhood cancer, have been the availability of drugs (more pronounced during regaining of independence), reaching new protocols and twinning for diagnosing rarer types of cancer.

All these improvements in diagnosing and treatment and changes in the health care organisation are reflected in survival outcomes. Starting from 23.8% in the 1970s, overall 5-year survival reached 73.0% by the latest period, which is not far below the European average for 2000–2007 (77.9, 95% CI 77.4–78.3) [2]. Survival increase has been relatively steady over time, but the biggest rise occurred in the 1990s.

The survival change in leukemias (from 7.5 to 83.8%) has been even more remarkable, being now equal to the rate observed in Finland for 2001–2010 [35]. Two leaps could be seen in leukemia survival. First from 1985 to 1989 (25.4%) to 1990–1994 (51.3%), coinciding with the introduction of first treatment protocols and the second from 1995 to 1999 (52.0%) to 2000–2004 (65.2%), when HDMTX treatment and ABMT became available. Last increase in survival rates can be associated with the access to registry donors for ABMT, which seemed to have a stronger impact primarily on AML survival. But looking at the two latest periods, 2005–2009 and 2010–2016, we can see that survival is plateauing (from 81.6 to 83.8%) (seen for other sites and overall survival as well, data not shown), indicating that the current treatment methods are depleting and new biology-driven approaches are needed [36]. The gap in survival between ALL and AML has narrowed over the study period, a trend that has also been shown in Switzerland and Finland [35, 37]. Biggest increments in 5-year survival increase for lymphomas were seen from 1990 to 1994 (49.9%) to 1995–1999 (70.3%) (data not shown), most likely due to the addition of HDMTX which has been shown to decrease relapse rate, especially for advanced disease [38]. Survival outcome dependency on methotrexate dose has been described for non-Hodgkin lymphomas [39, 40], which showed the biggest improvements in Estonia.

The largest improvements for CNS malignant tumors occurred from the 1980s to the 1990s, coinciding with the newly available treatment options and continued into the 2000s, reflecting treatment centralization. However, there has been a recent decline in CNS tumour survival as the latest estimate of 49.5% is well below the European rate of 57.5% for 2000–2007 [2] and Finnish rate of 59.5% in 2001–2010 [35]. As shown in previous analysis, the survival of all CNS tumour patients (including benign and borderline tumors) was 70.9% in Estonia in 2010–2014 [9], compared to 79.1% in Finland in 2001–2010 [35]. The survival estimates for CNS tumours may be somewhat affected by the use of different diagnostic methods and the classification of tumour behaviour [23]. As the number of unspecified cases has decreased, it is possible that some benign cases were previously recorded as malignant, thus included in the survival analysis and causing a higher survival estimate. It has been shown that the rate of unspecified cases was high for Estonia in earlier years [41]. The recent drop in survival could also be explained by changes in classification – pilocytic astrocytoma was downgraded to uncertain behaviour in ICD-O-3. As proposed by Stiller et al. [42] some cases of pilocytic astrocytoma could have been recorded as astrocytoma, NOS in earlier years, thus included in the analysis and causing a higher survival estimate. Our data shows a higher number of annual cases of astrocytoma, NOS in the first half of the study period and a higher number of pilocytic astrocytoma for the second.

Opposite to that shown for Northern-England [43], no significant changes in 5-year survival trends for neuroblastomas were observed in Estonia. Increase from 12.5% in the first decade to 47.4% in 1980–1989 marked the only substantial change in 5-year survival, which has stayed stable since. Even though the same pattern was shown for Finland [35], rates in Estonia have remained lower than those in Finland and Europe (for 2000–2010 40.0% in Estonia and 68.2% in Finland; for 2000–2002 72.0% in Europe) [35, 44]. Age > 18 months, male sex, metastasis at diagnosis and amplification of MYC-N are associated with poorer disease outcome [45]. Almost 60% of the cases diagnosed in Estonia were older than 18 months and with a M:F ratio of 1.3. Unfortunately, stage and tumor biology info was not available in registry data. A recent pilot study for the application of Toronto Staging confirmed the disease being diagnosed rather late, but not advanced, as majority of the cases were classified as stage L1 (local), giving a 3-year survival of 75% [46]. As for several other sites, low number of cases annually may also influence the result.

The huge gap in the survival of renal and malignant bone tumors between Estonian and Finnish patients in the 1980s has closed due to rapid improvements in Estonia throughout the 1990s and 2000s [35]. Hepatic tumors, the site group with one of the worst prognosis, is more prevalent in Eastern than in Western countries and seems to present a less favourable survival outcome for our region [15]. In Estonia, survival for hepatic neoplasms fluctuates greatly. Most of the cases were diagnosed in infants and specific symptoms generally develop late, but without data on stage, we can not conclude, whether this is the case. More detailed analysis could help to draw attention to possible prenatal parental exposures or shortcomings in treatment. Survival of 100% that could be seen from our data, for the period of 2010–2016, is most probably a random finding due to the small number of cases and does not reflect the current situation for hepatic malignancies. Germ cell tumors are a heterogenous group of tumors, presenting different rates of survival, depending on age and histology. Survival in Estonia reached 76.2% for 2000–2009, but then dropped to 37.0% in the latest period. The drop seen here is most probably random as low number of cases are more prone to cause a fluctuation in survival.

## Conclusion

Even though the societal and health care transition during the past decades have brought along major improvements in survival, childhood cancer patients in Estonia remain at a disadvantage. For some cancer sites, survival rates are still lower compared to many other European countries, and for others survival rates are plateauing. The small number of cases creates further challenges for diagnosis and care. Whereas the increasing incidence of some cancer sites may at least partly be due to improvements in diagnostic procedures and corresponding decreases in the incidence of unspecified neoplasms, overall cancer incidence in children is increasing. Thus, prioritization of childhood cancer by decision-makers to ensure access to standard care in both diagnosis and treatment, but also to innovative treatments is required to improve the outcomes of childhood cancer in terms of survival as well as the quality of survivorship. Continuous collection of high-quality data is crucial for monitoring progress, and the availability of more detailed clinical data would help to identify further possibilities for improvements in childhood cancer management in Estonia. Implementation of childhood cancer Toronto staging [47] system would also broaden the possibilities for more in depth survival analysis. The results are likely to be relevant for other countries in epidemiological and healthcare transition.

## Supplementary information

**Additional file 1: Table S1.** Annual percentage change in incidence by age groups and ICCC3 site-groups (malignant only), Estonia 1970–2016.

## Data Availability

Data can be made available upon reasonable request from the authors.

## Abbreviations

AAPC: Average annual percentage change
APC: Annual percentage change
DCO: Death certificate only
ECR: Estonian Cancer registry
ICCC-3: International Classification of Childhood Cancer, third edition
MV: Microscopically verified
