# Supplementary material for: Childhood cancer incidence and survival trends in Estonia (1970–2016): a nationwide population-based study
Source: BMC Cancer. 2020 Jan 10;20:30. doi: 10.1186/s12885-019-6510-7 (PMC6954517; doi:10.1186/s12885-019-6510-7)
Supplement: Supplementary file 1 — Additional file 1: Table S1. Annual percentage change in incidence by age groups and ICCC3 site-groups (malignant only), Estonia 1970–2016. [file 12885_2019_6510_MOESM1_ESM.pdf]

Supplementary Table 1. Annual percentage change in incidence by age groups and ICCC3 site-groups (malignant only), Estonia 1970–2016

	0–4 years		5–9 years		10–14 years	
	**APC (%)**	**95% CI**	**APC (%)**	**95% CI**	**APC (%)**	**95% CI**
**All sites**	0.7	-0.2–1.5	0.1	-0.5–0.7	**0.6**	**0.1–1.2**
**I Leukemias, myeloproliferative diseases, and myelodysplastic diseases**	0.0	-0.9–1.0	0.4	-0.5–1.2	0.6	-1.2–2.5
**II. Lymphomas and reticuloendothelial neoplasms**	1.0	-0.7–2.7	-1.6	-3.5–0.3	0.7	-0.9–2.3
**III. CNS and miscellaneous intracranial and intraspinal neoplasms (malignant only)**	1.5	-0.6–3.6	7.6[a]<br>-2.0	-2.3–18.6<br>-5.6–1.8	0.2	-1.6–2.0
**IV. Neuroblastoma and other peripheral nervous cell tumors**	**3.1**	**1.3–5.0**	-1.6	-4.4–1.2	-0.8	-11.7–11.5
**V. Retinoblastoma**	0.9	-2.6–4.5	NC		NC	
**VI. Renal tumors**	-1.1	-3.3–1.2	0.8	-4.4–6.2	NC	
**VII. Hepatic tumors**	2.4	-1.3–6.2	NC		NC	
**VIII. Malignant bone tumors**	NC		1.7	-4.2–8.0	-1.3	-4.1–1.6
**IX. Soft tissue and other extraosseous sarcomas**	1.6	-0.5–3.7	1.2	-3.5–6.1	1.0	-2.8–5.0
**X. Germ cell tumors, trophoblastic tumors, and neoplasms of gonads**	**2.7**	**0.4–5.1**	NC		1.2	-2.4–5.0
**XI. Other malignant epithelial neoplasms and malignant melanomas**	NC		NC		2.0	-0.4–4.4
**XII. Other and unspecified malignant neoplasms**	**-3.4**	**-5.9;-0.8**	NC		NC	

[a]joinpoint in 1985

NC not calculated, for sites with less than 10 cases over the study period

Statistically significant findings marked in bold
